# The gravistimulation-induced very slow Ca^2+^ increase in Arabidopsis seedlings requires MCA1, a Ca^2+^-permeable mechanosensitive channel

**DOI:** 10.1038/s41598-020-80733-z

**Published:** 2021-01-08

**Authors:** Masataka Nakano, Takuya Furuichi, Masahiro Sokabe, Hidetoshi Iida, Hitoshi Tatsumi

**Affiliations:** 1 Department of Biology, Tokyo Gakugei University, 4-1-1 Nukuikita-machi, Koganei, Tokyo 184-8501 Japan; 2 Research Institute for Science and Technology, Tokyo University of Science, 2641 Yamazaki, Noda, Chiba 278-8510 Japan; 3 Institute for Gene Research, Advanced Science Research Center, Kanazawa University, 13-1 Takaramachi, Kanazawa, Ishikawa 920-8640 Japan; 4 Faculty of Human Life Sciences, Hagoromo University of International Studies, 1-89-1 Hamadera-minamimachi, Sakai, Osaka 592-8344 Japan; 5 Mechanobiology Laboratory, Nagoya University Graduate School of Medicine, 65 Tsurumai, Nagoya, 466-8550 Japan; 6 Department of Applied Bioscience, Kanazawa Institute of Technology (KIT), 3-1 Yatsukaho, Hakusan, Ishikawa 924-0838 Japan

**Keywords:** Plant signalling, Abiotic

## Abstract

Gravity is a critical environmental factor affecting the morphology and function of plants on Earth. Gravistimulation triggered by changes in the gravity vector induces an increase in the cytoplasmic free calcium ion concentration ([Ca^2+^]_c_) as an early process of gravity sensing; however, its role and molecular mechanism are still unclear. When seedlings of *Arabidopsis thaliana* expressing apoaequorin were rotated from the upright position to the upside-down position, a biphasic [Ca^2+^]_c_-increase composed of a fast-transient [Ca^2+^]_c_-increase followed by a slow [Ca^2+^]_c_-increase was observed. We find here a novel type [Ca^2+^]_c_-increase, designated a very slow [Ca^2+^]_c_-increase that is observed when the seedlings were rotated back to the upright position from the upside-down position. The very slow [Ca^2+^]_c_-increase was strongly attenuated in knockout seedlings defective in MCA1, a mechanosensitive Ca^2+^-permeable channel (MSCC), and was partially restored in *MCA1*-complemented seedlings. The mechanosensitive ion channel blocker, gadolinium, blocked the very slow [Ca^2+^]_c_-increase. This is the first report suggesting the possible involvement of MCA1 in an early event related to gravity sensing in Arabidopsis seedlings.

## Introduction

Plants on Earth sense gravity and orient their growth direction with respect to the gravity vector, in a process known as gravitropism. Changes in the gravity vector generate a variety of intracellular signals, including changes in reactive oxygen species^1^, ionic gradient^2^, pH^3,4^, and cytoplasmic free calcium ion concentration ([Ca^2+^]_c_)^5–8^. Among these, the [Ca^2+^]_c_-increase is presumably involved in an early process of the gravitropic response^9,10^.

[Ca^2+^]_c_-increases in response to gravistimulation (typically turning a specimen in a vertical plane) have been reported repeatedly, e.g., [Ca^2+^]_c_-increases in maize coleoptiles were observed using a [Ca^2+^]_c_ indicator, fluo-3^11^. The [Ca^2+^]_c_-increases induced by gravistimulation have been studied in more detail in Arabidopsis (*Arabidopsis thaliana*) seedlings with aequorin, a luminous Ca^2+^-reporting protein^12–14^. When gravistimulated by turning from an upright to upside-down position, the seedlings showed a biphasic [Ca^2+^]_c_-increase in their hypocotyls and petioles^14^ imaged with an ultrasensitive photon counting camera; a fast-transient [Ca^2+^]_c_-increase and a subsequent slow [Ca^2+^]_c_-increase. The fast-transient [Ca2^+^]_c_-increase depended on the rotational velocity^13^ but not on the rotational angle, whereas the slow [Ca^2+^]_c_-increase depended on the rotational angle but not the rotational velocity^14^.

Recently, the slow [Ca^2+^]_c_-increase has been demonstrated as a gravistimulation-specific Ca^2+^-response using μg conditions produced by parabolic flights (PF)^13^; the μg conditions allowed the rotation of seedlings without gravistimulation, the fast-transient [Ca^2+^]_c_-increase was induced by rotation under the μg condition, and the slow [Ca^2+^]_c_-increase was initiated by transition from μg to ca. 1.5 g when the µ*g* condition was terminated^12,14^, confirming the idea that the fast-transient is principally induced by rotational stimulation, and the slow increase is genuinely induced by gravistimulation.

The slow [Ca^2+^]_c_-increase was significantly attenuated by the potential MSCC inhibitors Gd^3+^ and La^3+^, and the Ca^2+^ chelator 1,2-bis (2-aminophenoxy) ethane-N,N,N′,N′-tetraacetic acid (BAPTA), suggesting that it depends on the Ca^2+^ influx via MSCCs in the plasma membrane. The slow transient [Ca^2+^]_c_-increase was also attenuated by the actin-disrupting drugs cytochalasin B and latrunculin B^14^. These observations agree with the “starch-statolith model”^6,15,16^, which includes MSCCs and actin filament networks; sedimentation of “starch-statolith” increases the stress within the actin filament network and activates MSCCs^16^. However, the molecular entities of plasma membrane MSCCs responsible for sensing and responding to the gravistimulation have not been elucidated.

Recent studies identified five families of plant MS channels: the bacterial mechanosensitive channel of small conductance (MscS)-like proteins, MSLs^17,18^; Ca^2+^-permeable mechanosensitive channels (Mid1 complement activity), MCAs^19,20^ and two pore potassium channels, TPKs^21^, Piezos^22^ and reduced hyperosmolality-induced [Ca^2+^]_i_ increase (OSCA)^23^. Among these, MCAs, AtTPK4, and OSCA are plasma membrane MS cation channels^17,18,23–27^.

The functional properties of MCA1 have been examined in a variety of studies; Ca^2+^ uptake was increased in Arabidopsis seedlings and yeast cells by overexpressing MCA1 in the plasma membrane^19^. Seedlings of *mca1*-knockout lines lacked the ability to penetrate their roots into a harder agar^19^. Membrane stretching elevates [Ca^2+^]_c_ in CHO cells expressing MCA1^19^, and expression of Arabidopsis MCA1 leads to enhanced mechanosensitive cation channel activity (34 pS) in the *Xenopus laevis* oocyte plasma membrane^25^. Elongation growth of hypocotyls was suppressed under the hypergravity condition in wild-type Arabidopsis seedlings, whereas the extent of the suppression was reduced in *mca1*-knockout seedlings, but was augmented in *MCA1*-overexpressing seedlings^28,29^. These findings suggest that MCA1 is a plasma membrane Ca^2+^-permeable MS cation channel that is potentially involved in gravity sensing and the subsequent morphological changes in Arabidopsis seedlings.

In this study, [Ca^2+^]_c_-increases triggered by rotating Arabidopsis seedlings under 1–5 g conditions were investigated. With a backward rotation from an upside-down to an upright position, a novel, “very slow” [Ca^2+^]_c_-increase following the biphasic [Ca^2+^]_c_-increase was found. In addition, *mca1*-knockout seedlings showed a reduced amplitude of the very slow [Ca^2+^]_c_-increase, and the reduction was partially restored in *MCA1*-complemented seedlings. This is the first report that characterizes the very slow [Ca^2+^]_c_-increase by using 1–5 g gravitational acceleration, and the role of MCA1 in the gravistimulation-induced [Ca^2+^]_c_-increase.

## Results

### A novel very slow [Ca^2+^]_c_-increase induced by backward rotation from the upside-down to the upright position

**Figure 1:**
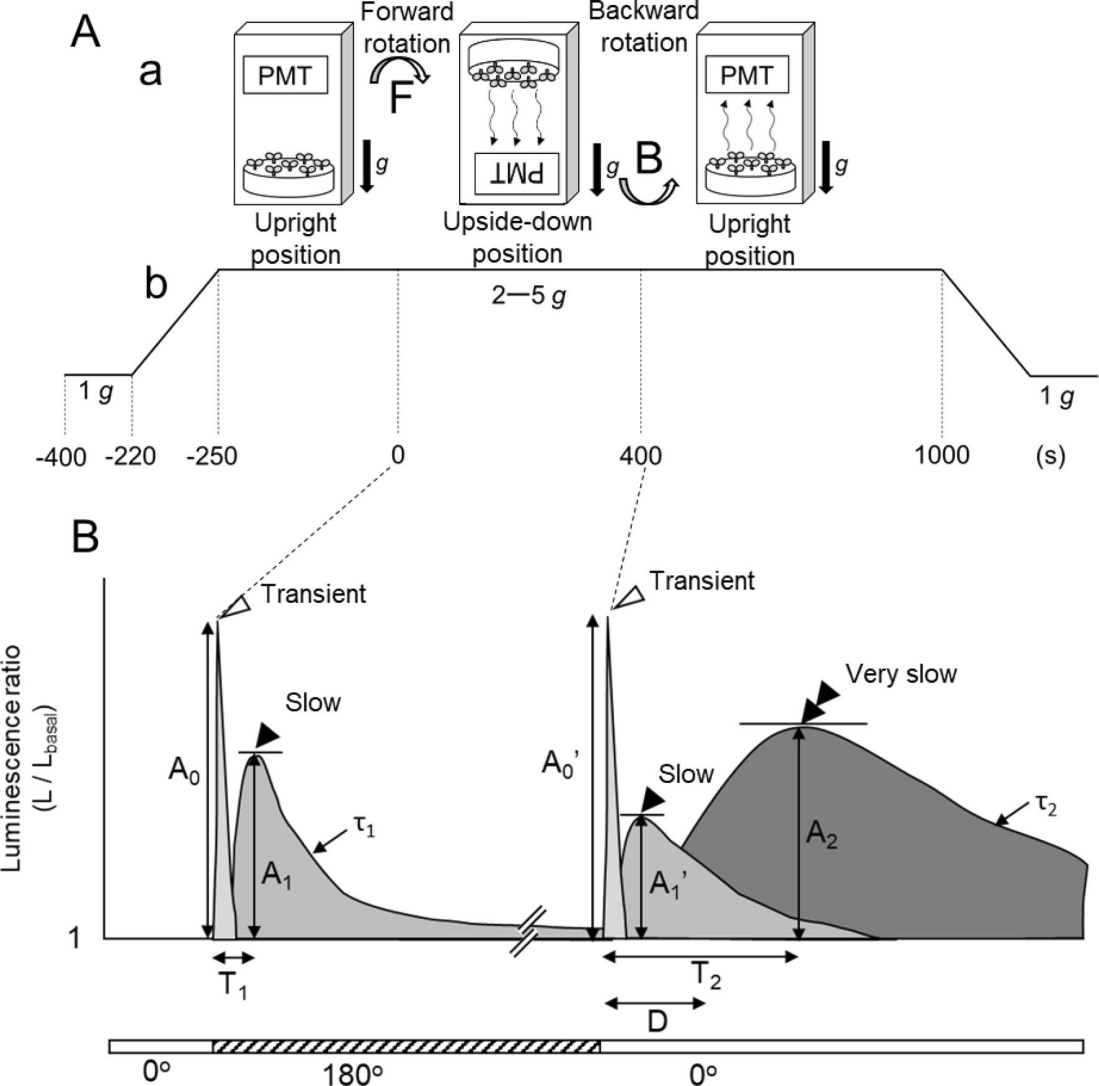
Diagram of the experimental protocol. **A** (**a**) Schematic diagrams of gravistimulation and detection of aequorin luminescence. A plate of Arabidopsis seedlings was mounted under a photomultiplier tube (PMT) in a light-tight dark box which was rotated by a stepping motor, enabling gravistimulation while monitoring the intensity of the aequorin luminescence. Changes in the direction of the Arabidopsis seedlings in the experimental protocol are illustrated. The arrow F shows the direction of forward rotation and B shows that of backward rotation. (**b**) Time course of the magnitude of gravitational acceleration applied to Arabidopsis seedlings. Seedlings were rotated + 180^o^ (forward rotation) at 0 s and − 180° (backward rotation) at 400 s. (**B**) Schematic illustration of a typical time course of changes in the luminescence ratio induced by gravistimulation. The peak of the transient [Ca^2+^]_c_-increase (light shading) is shown with a white arrowhead, the peak of the slow [Ca^2+^]_c_-increase (intermediate shading) with a black single arrowhead, and the peak of the very slow [Ca^2+^]_c_-increase (dark shading) with a black double arrowhead. The transient [Ca^2+^]_c_-increase (light shading) and the slow [Ca^2+^]_c_-increase (intermediate shading) are called biphasic [Ca^2+^]_c_-increases overall. Parameters (T_1_, T_2_, τ_1_, τ_2_, A_0_, A_0_′, A_1_, A_1_′, A_2_, and D) are shown and listed in Supplementary Table S1.

**Figure 2:**
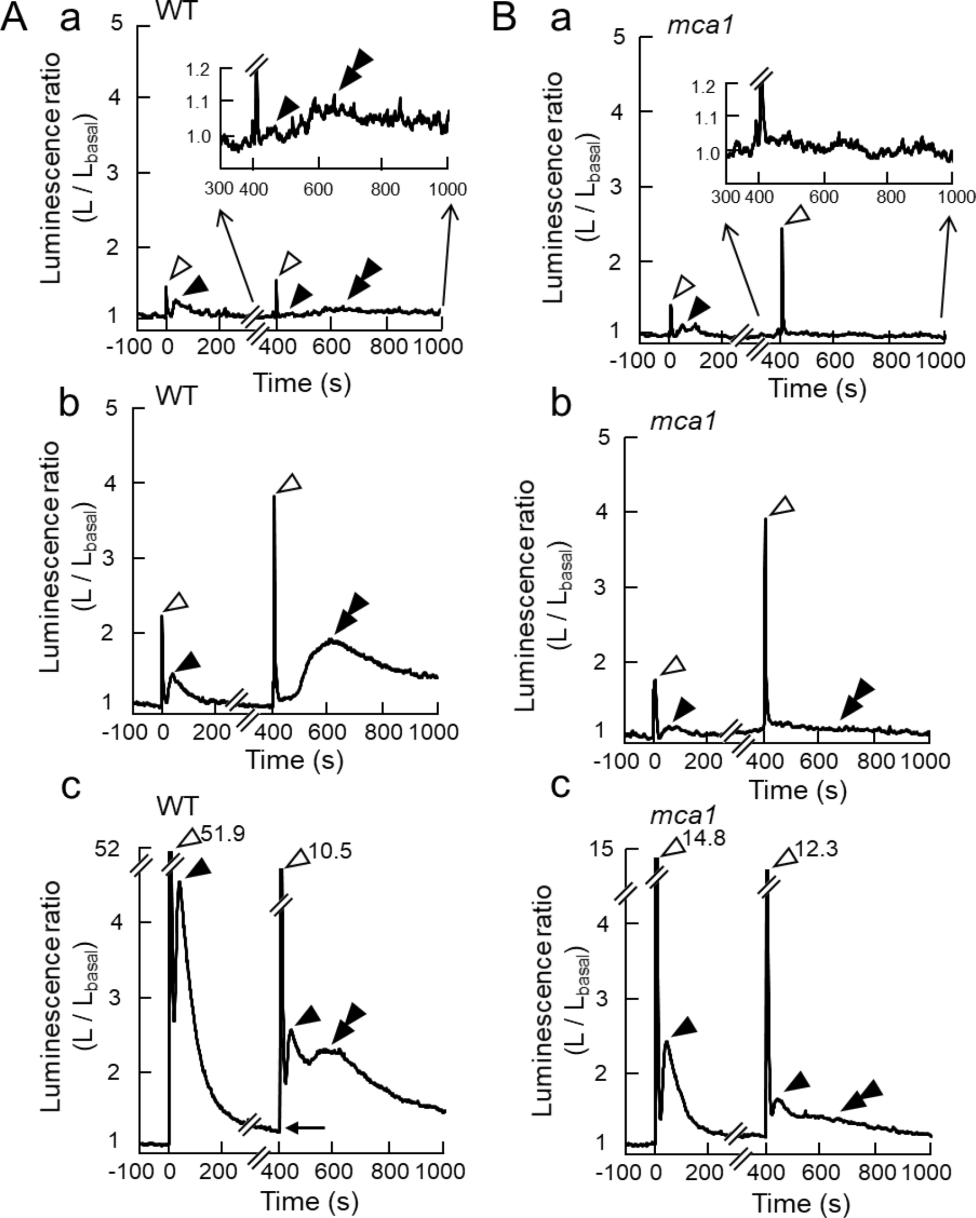
[Ca^2+^]_c_-increases induced by forward rotation to upside down and backward rotation to the upright position under 1, 3, and 5 g conditions. (**A**) a typical [Ca^2+^]_c_-increase in wild-type seedlings induced by forward rotation and backward rotation under 1 (panel **a**), 3 (panel **b**), and 5 g (panel **c**) conditions. (**B**) A typical [Ca^2+^]_c_-increase in an *mca1*-knockout mutant induced by rotations under 1, 3, and 5 g conditions. The very slow [Ca^2+^]_c_-increase is magnified and shown in the inset. Seedlings were rotated at 0 s and at 400 s. The notation is the same in panels A and B, and those of the arrowheads are the same as Fig. 1. Note the presence of the tail of the slow [Ca^2+^]_c_-increase at 400 s in panel Ac (horizontal arrow).

Arabidopsis seedlings containing aequorin were rotated to the upside-down position and then rotated back to the upright position under 1–5 g conditions (Fig. 1A), and gravistimulation-induced [Ca^2+^]_c_-changes were monitored. As described previously^14^, rotation to the upside-down position promoted a biphasic [Ca^2+^]_c_-increase, consisting of a fast-transient (white arrowhead with amplitude A_0_) and a subsequent slow [Ca^2+^]_c_-increase (black single arrowhead with amplitude A_1_) (The illustration in Fig. 1B; a fast-transient [Ca^2+^]_c_-increase and a subsequent slow [Ca^2+^]_c_-increases are shown by a white arrowhead and a black single arrowhead, respectively, and actual recordings are shown in Fig. 2A with the same notation).

**Figure 3:**
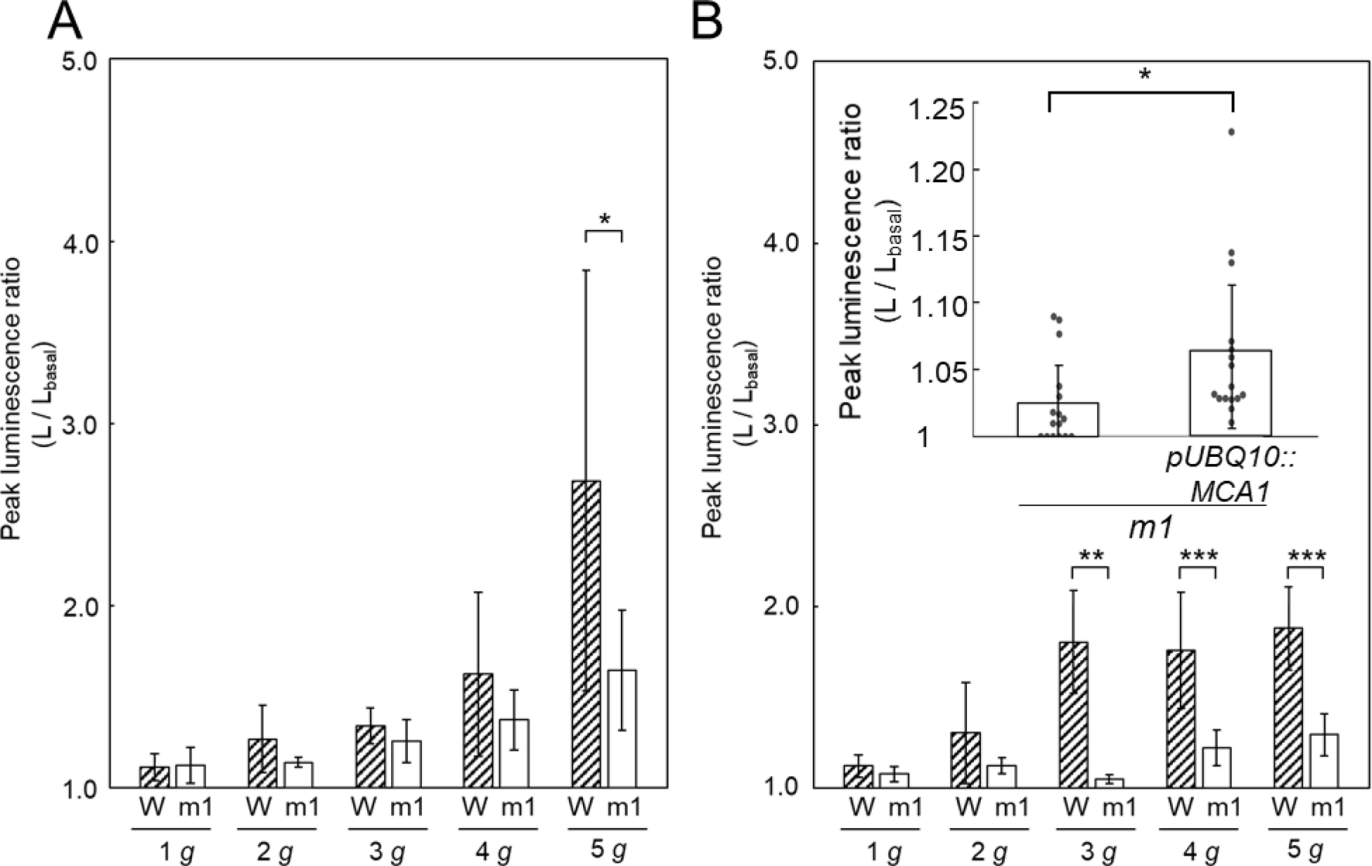
The peak amplitudes of the slow (**A**) and very slow [Ca^2+^]_c_-increases (**B**) induced by rotations under 1–5 g. Data represent means ± SD, and two tailed Student’s test was performed. Asterisks above the bars denote significant differences between the groups, and the peak amplitude of these responses are dependent on the magnitude of gravitational acceleration (*P < 0.05, **P < 0.01, ***P < 0.001). Letters W and m1 denote wild-type and *mca1-*knockout mutant seedlings, respectively. The inset in (**B**) shows the amplitude of the very slow [Ca^2+^]_c_-increase under 3 g conditions in an *mca1-*knockout mutant (left) and *MCA1*-complemented mutant (right) (*n* = 16).

Both the amplitude of the fast-transient and slow [Ca^2+^]_c_-increases were augmented by increasing the magnitude of the gravitational acceleration from 1 to 5 g (Figs. 2A, 3A). The slow [Ca^2+^]_c_-increase declined to the basal level with a decay time constant, τ_1_ = 76.4 s ± 20.9 s, *n* = 8 under 3 g (Supplementary Table S1), then backward rotation to the upright position was given at 400 s after the first rotation where the slow [Ca^2+^]_c_-increase almost disappeared (Fig. 1B). As described previously^14^, the backward rotation also promoted the biphasic Ca^2+^-signal with similar kinetics to that in the first rotation, but the amplitude of the second slow [Ca^2+^]_c_-increase was apparently attenuated, and often not observed (black single arrowhead with amplitude A_1_′ in Figs. 1B, 2Ab). The backward rotation additionally induced a very slow [Ca^2+^]_c_-increase (black double arrowhead with amplitude A_2_ in Figs. 1B, 2A). The very slow [Ca^2+^]_c_-increases peaked at 225 s on average after rotation (T_2_ in Fig. 1B), and very slowly decayed to the basal level (Fig. 2A; Supplementary Table S1).

The amplitudes of the fast transient (A_0_ in Fig. 1B), the slow [Ca^2+^]_c_-increases (A_1_ in Fig. 1B) induced by the forward rotation, and the very slow [Ca^2+^]_c_-increase (A_2_ in Fig. 1B) induced by the backward rotation were augmented with increasing the magnitude of the gravitational acceleration (Figs. 2A, 3A). In the case of the very slow [Ca^2+^]_c_-increase, the amplitude of the response was almost saturated at 3 g acceleration (nearly the same peak amplitudes of [Ca^2+^]_c_-increase were detected in 3–5 g, Fig. 3B), whereas the amplitude of the slow [Ca^2+^]_c_-increase was augmented, depending on the *g* magnitude; the saturation was not seen in the range from 1 to 5 g (Fig. 3A). Thus, the *g* dependency of the slow and the very slow [Ca^2+^]_c_-increases is not the same.

The kinetics parameters of the slow and very slow [Ca^2+^]_c_-increases were clearly different. The time to peak (T_2_ in Fig. 1B) of the very slow [Ca^2+^]_c_-increase was longer than that of the slow [Ca^2+^]_c_ increase (T_1_ in Fig. 1B). The delay from the time of rotation to the time of initial rising phase of the very slow [Ca^2+^]_c_-increase (D in Fig. 1B) was estimated at 90.0 s ± 10.0 s (*n* = 5 under 3 g), which is approximately five times longer than the delay of the onset of the slow [Ca^2+^]_c_-increase (18 s) in the previous study. The time constant of the decay of the very slow [Ca^2+^]_c_-increase (τ_2_) was also longer than that of the slow [Ca^2+^]_c_-increase (τ_1_) (see Supplementary Table S1). Time to the peak (T_1_ and T_2_ in Fig. 1B) and time constant of the decay (τ_1_ and τ_2_ in Fig. 1B) were not affected by the changes in gravitational acceleration (1–5 g) (Supplementary Table S1).

**Figure 4:**
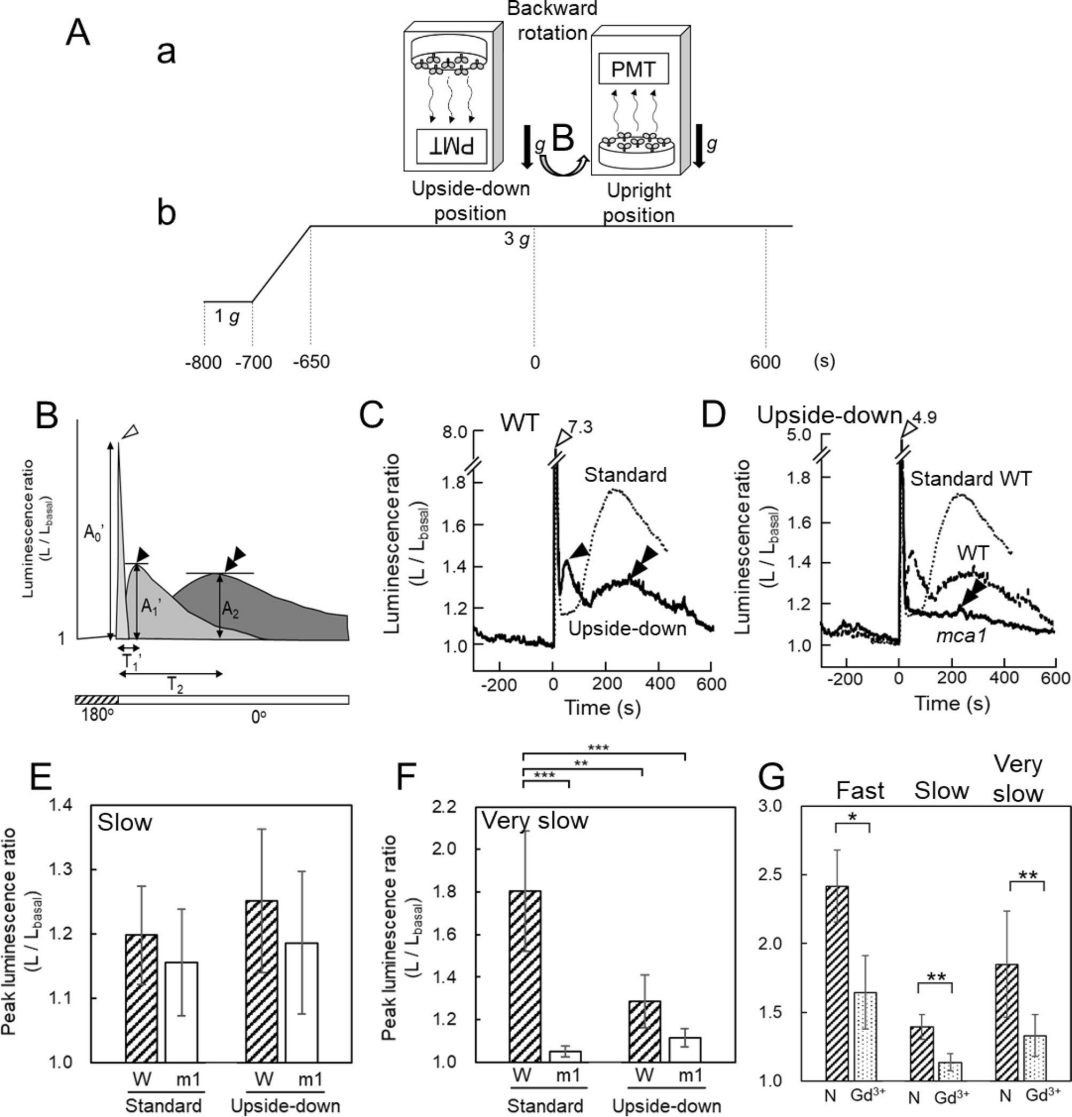
The very slow [Ca^2+^]_c_-increase induced by backward rotation to the upright position in pre-rotated plants. **A** (**a**) The direction of Arabidopsis seedlings in the experimental protocol. (**b**) Time course of the magnitude of gravitational acceleration. The seedlings used for this experiment were kept in an upside-down position for 2–4 h prior to the experiment. (**B**) Schematic illustration of the time course of the change in the luminescence ratio induced by gravistimulation (backward rotation). The notation is the same as Fig. 1. (**C**) A typical [Ca^2+^]_c_-increase induced by backward rotation under 3 g conditions in the wild-type seedlings, and the dotted lines show the mean time course of the slow and very slow [Ca^2+^]_c_-increases by the standard protocol. (**D**) A typical [Ca^2+^]_c_-increase in *mca1*-knockout mutant seedlings induced by backward rotation under 3 g conditions, and the dotted lines shows the mean time course of the slow and very slow [Ca^2+^]_c_-increases of preconditioned wild-type plants, and the time course of wild-type [Ca^2+^]_c_-increase by standard protocol. (**E**) The amplitudes of the slow [Ca^2+^]_c_-increase induced by backward rotation under 3 g conditions (A_1_′). W: wild-type, m1: *mca1* knockout, standard: the standard protocol, upside-down: the protocol shown in the panels A and B (seedlings were pre-rotated 2–4 h before the experiment). (**F**) The amplitudes of the very slow [Ca^2+^]_c_-increase induced by backward rotation under 3 g conditions. (**G**) The amplitudes of fast [Ca^2+^]_c_-increases (A_0_, “Fast”), slow [Ca^2+^]_c_-increases (A_1_, “Slow”), and very slow [Ca^2+^]_c_-increase (A_2_, “Very slow”) induced by backward rotation under 3 g conditions were decreased by Gd^3+^ (100 μM). Data represent the mean ± SD. *n* = 3. Data represent mean ± SD. *n* = 4. *P < 0.05, **P < 0.01, two-tailed Student’s test.

The [Ca^2+^]_c_-increase in *mca1*-knockout seedlings induced by the same stimulation protocol showed that the amplitudes of the very slow [Ca^2+^]_c_-increases were profoundly decreased (Figs. 2B, 3, 4D,F). The [Ca^2+^]_c_-increase in *MCA1*-complemented *mca1*-knockout seedlings induced by the same stimulation protocol showed that the amplitude of the very slow [Ca^2+^]_c_-increase by backward rotation was partially restored under 3 g condition (Fig. 3B inset). The kinetic parameters (T_1_, T_2_, τ_1_, τ_2_) and the amplitudes of the fast transients (A_0_, A_0_′) were not changed apparently in *mca1*-knockout seedlings as shown in Supplementary Table S1. Note that the slow [Ca^2+^]_c_-increase was also affected to a lesser extent under 1–4 g, but the difference between WT and *mca1* mutants became apparent under 5 g, suggesting the slow [Ca^2+^]_c_-increase induced by 180° rotation under 5 g conditions includes activation of MCA1 to some extent.

The amplitude of the slow [Ca^2+^]_c_-increases and very slow [Ca^2+^]_c_-increases under 3 g was decreased to 34% and 39% of wild-type seedlings, respectively, by the presence of MSCC inhibitors Gd^3+^ (100 μM; Fig. 4G). These data suggest that MCA1, a mechanosensitive cation channel in the plasma membrane, is involved in the gravistimulation-induced [Ca^2+^]_c_-increases.

### The very slow [Ca^2+^]_c_ increase was attenuated when plants were preconditioned to the upside-down position

Upside-down positioning for 2–4 h was long enough to detect PIN3 transcytosis to the bottom side of the cell, and redirect the auxin flux to generate asymmetric auxin accumulation^30^. In this context, plants would be in part adapted to the upside-down positioning, which may affect both the slow and very slow [Ca^2+^]_c_-increase induced by the backward rotation. Arabidopsis seedlings were preconditioned to the upside-down position for 2–4 h to familiarize them with the upside-down position, then rotated backward under 3 g (the protocol is shown in Fig. 4A). The amplitude of the slow [Ca^2+^]_c_-increase induced by backward rotation was in the same range as that induced by the first rotation, as shown in Figs. 4B,C (black single arrowhead) and Fig. 4E. In contrast, the amplitude of the very slow [Ca^2+^]_c_-increase was profoundly reduced, as shown in Fig. 4B,C (black double arrow head) and summarized in Fig. 4F (A_2_ declined to 36% of the control *n* = 4, under 3 g), suggesting again that the slow and very slow [Ca^2+^]_c_-increases were affected in a different way by prepositioning in the upside-down position. The reduction of the very slow [Ca^2+^]_c_-increases may reflect the adaptation of plants to the upside-down positioning. The mean time course of the slow and very slow [Ca^2+^]_c_-increases by the standard protocol is superimposed on the [Ca^2+^]_c_-increase of the preconditioned WT plants (Fig. 4C,D), and a typical time course of the slow and very slow [Ca^2+^]_c_-increases of preconditioned plants is superimposed on that of *mca1* mutants (Fig. 4D) to show up the inhibitory effect of the precondition on the [Ca^2+^]_c_-increase.

The tail of the slow [Ca^2+^]_c_-increase slightly overlaps the initial part of the very slow [Ca^2+^]_c_-increase (see the horizontal arrow in Fig. 2Ac), which may affect the kinetics of the very slow [Ca^2+^]_c_-increase. The kinetic parameters of this very slow [Ca^2+^]_c_-increase in prepositioned plants (there was no overlap) were nearly the same as those of the very slow [Ca^2+^]_c_-increase induced by the standard protocol (with overlap), implying the tail of the slow [Ca^2+^]_c_-increase does not affect the kinetics of the very slow [Ca^2+^]_c_-increase. The [Ca^2+^]_c_-increase in *mca1*-knockout seedlings showed that the amplitudes of the very slow [Ca^2+^]_c_-increases were profoundly decreased by the preconditioned protocol (Fig. 4D).

### Kinetic parameters of the very slow [Ca^2+^]_c_-increase were not affected by a sudden decrease in gravitational acceleration from 3 to 1 g

The PF experiment revealed that when gravistimulation triggered the response, a sudden gravitational decrease from 2 g to µg did not attenuate the slow [Ca^2+^]_c_-increase; i.e., the response was not affected by the sudden *g* decrease. This indicates that once the gravity sensing process is activated, the slow [Ca^2+^]_c_-response proceeds irrespective of environmental gravity changes.

**Figure 5:**
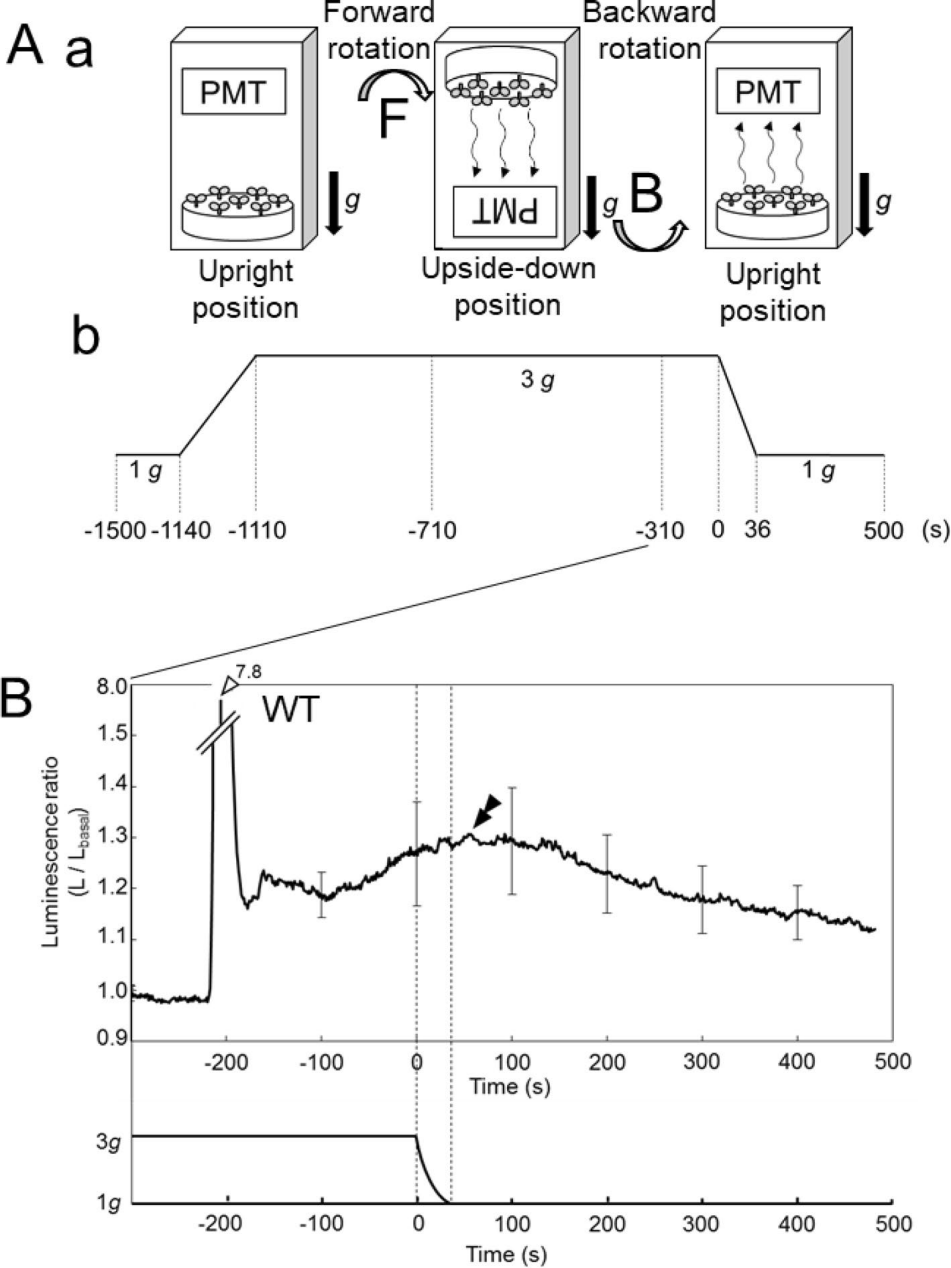
The very slow [Ca^2+^]_c_-increase induced by backward rotation was not affected by a decrease in the gravitational acceleration from 3 to 1 g. (**A**) diagram of the experimental protocol. (**B**) The averaged trace of changes in the luminescence ratio in response to gravistimulation. Gravitational acceleration decreased from 3 to 1 g in 36 s. Data represent means ± SD. *n* = 8.

The amplitude of the centrifugation was reduced from 3 to 1 g and the time course of the very slow [Ca^2+^]_c_-increase of WT plants was examined (Fig. 5A); the time course was not changed apparently by the 3–1 g sudden decline, as shown in Fig. 5B, suggesting that once the gravity sensing process is activated, the very slow [Ca^2+^]_c_-response proceeds irrespective of environmental gravity changes.

## Discussion

In the present study, the [Ca^2+^]_c_ changes in response to changes in the gravity vector under hypergravity conditions up to 5 g were examined. The backward rotation from the upside-down to upright positions induced a very slow [Ca^2+^]_c_-increase. The very slow [Ca^2+^]_c_-increase was profoundly diminished in *mca1*-knockout seedlings and was partially restored in *MCA1*-complemented seedlings, suggesting a possible involvement of the Ca^2+^-permeable plasma membrane mechanosensitive channel MCA1 in this gravistimulation-induced [Ca^2+^]_c_-increase in Arabidopsis seedlings. Kinetic analyses of the very slow [Ca^2+^]_c_-increase suggest that the molecular mechanism behind the very slow [Ca^2+^]_c_-increase is distinct from that of the slow [Ca^2+^]_c_-increase.

Once the very slow [Ca^2+^]_c_-increase is activated, the [Ca^2+^]_c_ response proceeded irrespective of gravity changes. The time course of the very slow [Ca^2+^]_c_-increase was not changed when the gravitational acceleration declined from 3 to 1 g at the peak of the response, which agrees with the hypothesis that gravistimulation produced by the backward rotation leads to the activation of the plasma membrane MSCCs (e.g., MCA1), followed by a molecular interaction and signaling process that were not affected by gravity changes.

Our previous pharmacological study using Ruthenium Red suggested the involvement of endomembrane Ca^2+^-permeable channels in the slow [Ca^2+^]_c_-increase. The intracellular inositol 1,4,5-trisphosphate (InsP_3_) level is known to increase within 15 s of gravistimulation in oat shoot pulvini, suggesting the possible involvement of phospholipase C (PLC)-dependent signaling. Thus, once the gravity sensing process is triggered, it can be followed by a signaling process including the activation of PLC, production of InsP_3_, and InsP_3_-induced Ca^2+^-release (IICR) from the ER (or the vacuole), which may be less sensitive to changes in the gravity environment. This explains why the kinetic parameters of the slow and very slow [Ca^2+^]_c_-increases were not affected by the different magnitudes of gravitational acceleration (Supplementary Table S1); i.e., the slow kinetics may be predominantly governed by the Ca^2+^ release processes from cellular organelles^13,31^.

Enhanced gravitropism by hypergravity (hyper-gravitropism) is reported in Arabidopsis roots^32^, stems^33^ and hypocotyls^34^. The analysis of the plant growth under the sequential hypergravity conditions (30–500 g for 1 day) shows that hypergravity suppressed elongation growth of hypocotyls, but this effect was reduced in hypocotyls of *mca*-null mutants compared with the wild type^28^, suggesting that MCAs are involved in the sensing of gravity signals in plants. Hyper-gravitropism was not examined in this study, since it may need very high *g* condition and long observation period according on the above study.

The fast-transient [Ca^2+^]_c_-increase was presumably a genuine response to rotation, because it was induced by rotatory stimulation even under µ*g*, and its amplitude increases in an angular acceleration-dependent manner. Recently, it was demonstrated that the amplitude of the fast-transient [Ca^2+^]_c_-increase (A_0_ and A_0_′) is augmented by the gravitational acceleration from μg to 2 g. The present study confirmed these results under a wider range of gravitational acceleration, from 1 to 5 g. In contrast, the amplitude of the slow [Ca^2+^]_c_-increase depended on the rotational angle (change in the direction of gravity)^12,14^, not on the rotational velocity, and it is a genuine *g*-change response. These support the idea that the backward rotation induces fast transient, slow and very slow [Ca^2+^]_c_-increases as illustrated in Fig. 1, however, the possibility is not excluded that further investigation reveals additional complexity in the [Ca^2+^]_c_-increase.

Hyper-gravistimulation, achieved by reorienting the specimens 90° under the hypergravity condition (e.g., 5 g), enhances gravitropic curvature in Arabidopsis hypocotyls and roots^32^, whereas gravistimulation less than 1 g (e.g., 0.39–0.93 g), created by centrifugation in space, reduced gravitropic curvature in lentil roots^35^. These observations are consistent with the gravity-dependency of the slow and very slow [Ca^2+^] _c_-increases in 1–5 g conditions. Thus, plants that have evolved on earth (1 g) may be capable of transducing a wide range of gravitational changes into the gravitropic response by potentiating the amplitude of Ca^2+^-signaling.

Our results suggest that plants use MCA1 for detecting the gravity vector from an upside-down to upright position, i.e., changes from an unfavorable position to their accustomed position. On the other hand, plants may use multiple molecular machineries for sensing gravity vector changes (e.g., upright to upside-down position). This may be the reason why the amplitude of the slow [Ca^2+^]_c_-increase was less affected in *mca1*-knockout seedlings, i.e., multiple MSCCs species may be responsible for the slow [Ca^2+^]_c_-increase, and *mca1*-knockout may only partially affect the slow [Ca^2+^]_c_-increase (e.g., 3 g condition).

The characteristics of the [Ca^2+^]_c_-increase are consistent with the starch-statolith hypothesis: sedimentation of the high-density plastid, and amyloplast in response to the gravity vector, increase stress in the actin filaments, which may activate mechanosensitive Ca^2+^-permeable channels (e.g., MCA1)^16^. This idea is supported by the experimental result that mechanical stress in the actin cytoskeleton can activate MS channels^36^. A centrifuge microscope revealed sedimentary movements of amyloplasts under hypergravity conditions^33^. In this study backward rotation specifically activated MCA1, leading to the very slow [Ca^2+^]_c_-increase, implying that the plant distinguishes the forward and backward rotation. At present the cellular mechanism that distinguishes the forward and backward rotation is not known, however, it might be worth to propose a hypothetical cellular mechanism behind this. The forward rotation will cause the sedimentation of the high-density plastid, which will increase stress in the certain set of actin filaments that is newly encountered with the plastid, and the stress increase in the actin filaments will activate multiple mechanosensitive Ca^2+^-permeable channels including MCA1. On the other hand, the backward rotation will also cause the sedimentation, but this may increase stress in the “different” set of actin filaments that was used to be associated with the plastid under the upright position before gravistimulation and are primarily connected with MCA1, and the stress increase causes the very slow [Ca^2+^]_c_-increase.

MCA2 is the only paralog of MCA1 in Arabidopsis. Ca^2+^ uptake activity was lower in the roots of *mca2*-null plants than those of wild-type plants, and overlapping function of MCA1 and MCA2 in gravitropism is reported^20^. However, the gravistimulation induced Ca^2+^ response in MCA2 knockdown mutant was not examined in this study, because the [Ca^2+^]_c_ increases are recorded in hypocotyls and petioles of seedlings but not in roots in our experimental condition (coelenterazine-CP does not penetrate the gel substrate).

The physiological role of the very slow [Ca^2+^]_c_-increase has not been elucidated at present. The Ca^2+^ signals were collected from hypocotyls and petioles as mentioned in our preceding report^14^, which shows negative gravitropism. When plants are temporarily rotated and then rotated back to the upright position, plants should terminate the gravitropic responses (e.g., auxin signaling in hypocotyls) induced by the temporal rotation. If the very slow [Ca^2+^]_c_-increase might be involved in the termination of gravitropic responses, it affect the Ca^2+^-related relocalization of PIN auxin transporter proteins or modulate auxin-dependent growth^37–39^. These ideas should be examined in future studies.

## Materials and methods

### Plant materials, growth conditions, and reconstitution of aequorin

The materials and methods of this study are basically the same as those used in our previous study^14^. Briefly, approximately 50 *Arabidopsis thaliana* (Col-0) seeds expressing cytoplasmically targeted apoaequorin were surface-sterilized, then sown on an agar plate containing plant growth medium (MS medium containing 1 × Murashige and Skoog salts, 1% [w/v] Suc, 0.01% [w/v] *myo*-inositol and 0.05% [w/v] MES pH 5.8, adjusted with 1 M KOH, solidified with 0.3% gelrite) in a petri dish (diameter, 6 cm), and incubated at 22 °C in a growth chamber under continuous white light for 7–8 days after stratification at 4 °C in the dark for 2 days. These seedlings were incubated with the reconstitution medium (3 mL of liquid MS medium containing 2.5 μM coelenterazine-CP) for approximately 16 h at 22 °C in darkness to reconstitute aequorin. The liquid medium was then removed from the plate 2–6 h prior to the experiment.

### Construction of *mca1*-knockout and *MCA1*-complemented mutants expressing aequorin

The *mca1*-knockout and *MCA1*-complemented mutant lines were described previously^19^. *mca1*-knockout and *MCA1*-complemented mutant lines expressing apoaequorin were made by transformation with the strong *UBIQUITIN10* promoter of Arabidopsis^40^. The apoaequorin-coding sequence was cloned into the Gateway entry vector pDONR by PCR amplification, and recombination of the apoaequorin construct into the expression vector pUB-DEST was conducted using the LR Gateway system^41^. The resulting pUB-DEST-apoaequorin construct was introduced into *Agrobacterium tumefaciens* (GV3101) for transformation of *mca1*-knockout and *MCA1*-complemented mutant lines by the floral dip method^42^. Transgenic plants expressing apoaequorin under the control of the *UBIQUITIN10* promoter were selected using BASTA selective medium and were checked by PCR. Homozygous apoaequorin-expressing T3 and T4 plants were used for analysis.

### [Ca^2+^]_c_ monitoring and a device for gravistimulation

A seedling-growing plate was mounted below a photomultiplier tube (PMT, model H7828, Hamamatsu Photonics). [Ca^2+^]_c_-dependent aequorin luminescence was monitored and processed using a photon counter (model SUC-100, SCIENTEX Co.) at 1-s intervals. This configuration enabled the detection of gravistimulation-induced aequorin luminescence generated from approximately 50 seedlings. Four sets of this configuration were used for the experiment; each set was mounted in a light-tight dark box and aequorin luminescence was recorded and stored on a computer. This box was vertically rotated 180° by a computer-controlled DC motor system (model BX5120AM-100S, ORIENTAL MOTOR Co.) at an angular velocity of 6 rpm and an angular acceleration of 2.5 rad s^−2^, because it induced nearly the maximum [Ca^2+^]_c_-increase compared with that by 90° rotation^14^. The plates were mounted near the rotation axis, producing a centrifugal acceleration of ca. 10^–3^ g during uniform circular motion; the tangential acceleration at the start of rotation was ca. 10^–2^ g. These devices were mounted on a custom-made aluminum rack that was fixed on the bucket of a swing type 5 g centrifuge machine (W3 experimental unit, Japan Aerospace Exploration Agency, Japan, Supplementary Fig. S1). Measurements were made when the experimental setup reached a constant acceleration (2–5 g). The amplitude and kinetic parameters of the fast-transient, slow, and very slow [Ca^2+^]_c_-increases were analyzed, as described below. Gravitational acceleration applied to the specimen was monitored using an accelerometer (model CXL04LP3, Crossbow Technology, Inc.) and stored on a computer. The complementation test was conducted as a separate experiment following the WT and *mca1* mutant experiments.

### Treatment with chemical agents

GdCl_3_ (10 mM stock) was added to a final concentration of 100 µM to the aequorin-reconstitution medium in a petri dish 1 h prior to the removal of the medium. Plant growth medium containing coelenterazine-CP and GdCl_3_ was removed from the dish 30 min before the experiment^14^ to retain the inhibitory action of Gd^3+^ during the experiment.

### Data analyses

In this paper, the luminescence ratio, rather than the calibrated [Ca^2+^]_c_, was used for all analyses, because it was not possible to discharge the remaining aequorin and monitor the signal in the 5 g centrifuge machine. The luminescence ratio was calculated by dividing the aequorin luminescence intensity by the basal luminescence intensity; the average aequorin luminescence intensity before the first gravistimulation at the gravitational acceleration for each experiment (i.e., − 200 to − 50 s in Fig. 1A). The Ca^2+^ response showed statistical nature; e.g., the amplitude of the fast transient, the slow and very slow Ca^2+^ increases varied from response to response. Data were analyzed using two-tailed Student’s *t* test or one-way ANOVA statistical analysis with the software Origin version 9, which was also used to estimate the decay time constant. The number of observations (*n*) denotes the number of experiments made with independent samples/plates containing approximately 50 seedlings. All experiments were repeated more than three times, and all the data obtained were analyzed.

## Supplementary Information

Supplementary Information

## Data Availability

*Accession numbers* MCA1 Gene ID: 829747.

## Abbreviations

[Ca^2^^+^]_c_: Cytoplasmic free calcium ion concentration
MCA1: *mid1*-Complementing activity 1
PFs: Parabolic flights
MSCC: Mechanosensitive Ca^2^^+^-permeable channel
IICR: InsP_3_-induced Ca^2^^+^-release
InsP_3_: Inositol 1,4,5-trisphosphate
MscS: Mechanosensitive channel of small conductance
MS: Mechanosensitive
