# Supplementary material for: The gravistimulation-induced very slow Ca2+ increase in Arabidopsis seedlings requires MCA1, a Ca2+-permeable mechanosensitive channel
Source: Sci Rep. 2021 Jan 8;11:227. doi: 10.1038/s41598-020-80733-z (PMC7794229; doi:10.1038/s41598-020-80733-z)
Supplement: Supplementary file 1 — Supplementary Information [file 41598_2020_80733_MOESM1_ESM.docx]

Supplementary Information

**The gravistimulation-induced very slow Ca^2+^ increase in Arabidopsis seedlings requires MCA1, a Ca^2+^-permeable mechanosensitive channel**

Masataka Nakano^1, 2, 3^, Takuya Furuichi^4^, Masahiro Sokabe^5^, Hidetoshi Iida^1^, and Hitoshi Tatsumi^6^

----------------------------------------------------------------------------------------------------------

^1^ Department of Biology, Tokyo Gakugei University, 4-1-1 Nukuikita-machi, Koganei, Tokyo 184-8501, Japan

^2^ Research Institute for Science & Technology, Tokyo University of Science, 2641 Yamazaki, Noda, Chiba 278-8510, Japan

^3^ Institute for Gene Research, Advanced Science Research Center, Kanazawa University, 13-1 Takaramachi, Kanazawa, Ishikawa, 920-8640, Japan

^4^ Faculty of Human Life Sciences, Hagoromo University of International Studies, 1-89-1 Hamadera-minamimachi, Sakai, Osaka 592-8344, Japan

^5^ Mechanobiology Laboratory, Nagoya University Graduate School of Medicine, 65 Tsurumai, Nagoya 466-8550, Japan

^6^ Department of Applied Bioscience, Kanazawa Institute of Technology (KIT), 3-1 Yatsukaho, Hakusan-shi, Ishikawa 924-0838 Japan

Correspondence and requests for materials should be addressed to H. T. (e-mail: [tatsumi@neptune.kanazawa-it.ac.jp](mailto:tatsumi@neptune.kanazawa-it.ac.jp))

**
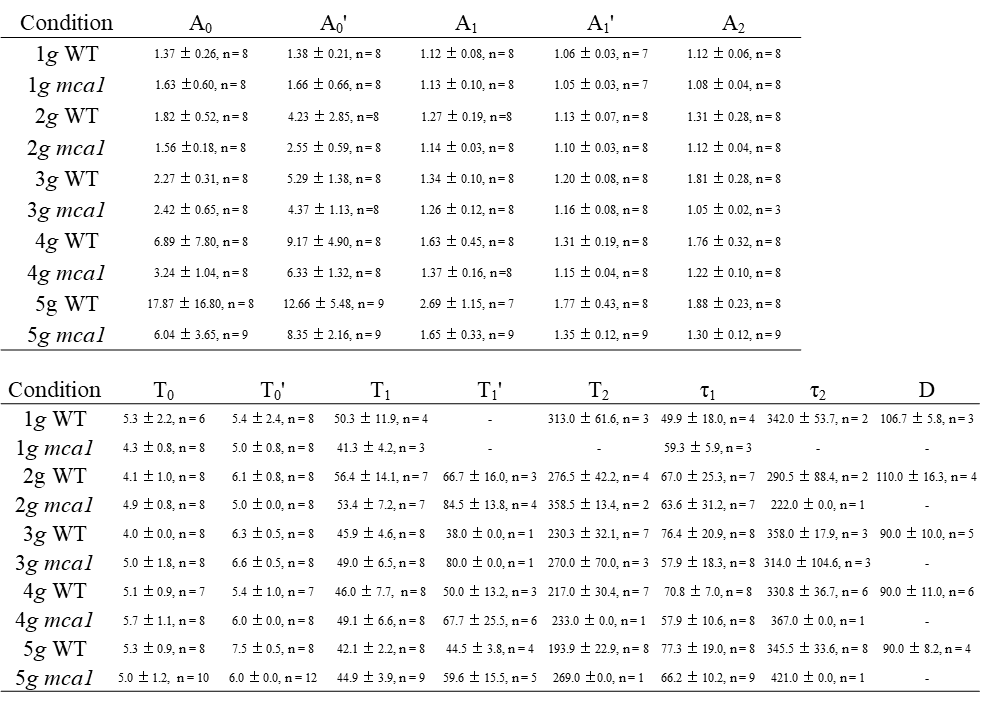
**

**Table S1.** Kinetic parameters (A_0_, A_0_’, A_1_, A_1_’, A_2_, T_0_, T_0_’, T_1_, T_2_, τ_1_, τ_2_, D) of the [Ca^2+^]_c_-increase induced by rotations under different gravitational conditions. All parameters measured in this study are shown in Fig. 1C. Data represent the mean ± S.D. τ_1_ and τ_2_ are the time constant of the decay. D denotes the delay from the onset of rotation to the initial rising phase of the very slow [Ca^2+^]_c_-increase. No significant difference by increasing the gravitational acceleration was detected in T_1_ between WT and *mca1* seedlings with one-way ANOVA. No significant difference was found for T_2_, and τ1 and τ2 with the same analysis.


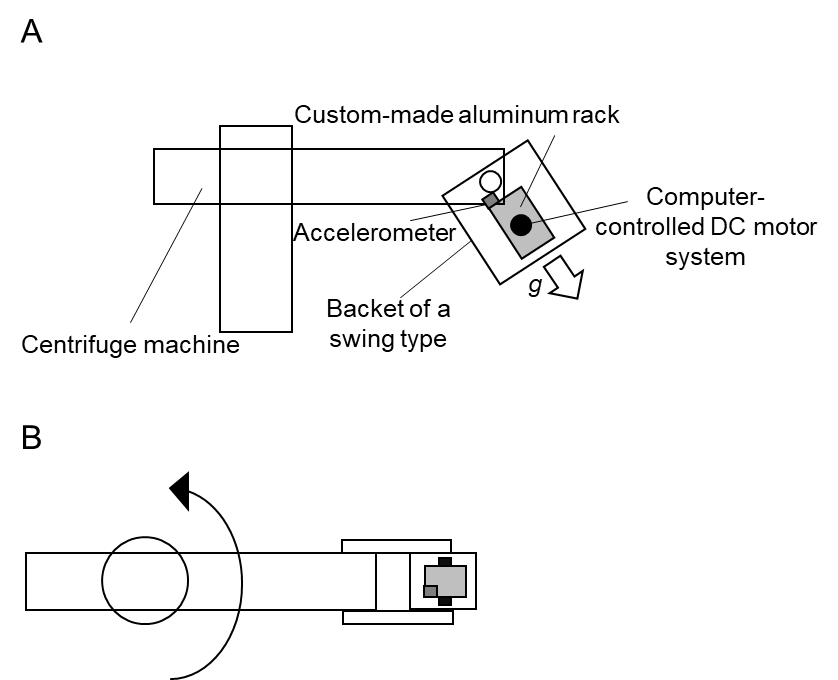


**Figure S1.** Schematic diagram of the experimental device. A, Side view. B, Top view.
